# From maintenance to modulation: Rethinking the role of DNA methyltransferase 1 in neuronal development

**DOI:** 10.4103/NRR.NRR-D-25-01223

**Published:** 2026-02-05

**Authors:** Geraldine Zimmer-Bensch

**Affiliations:** RWTH Aachen University, Department of Neuroepigenetics, Institute of Zoology (Biology 2), Aachen, Germany

The study by Reichard et al. (2025), entitled “DNMT1-mediated regulation of somatostatin-positive interneuron migration impacts cortical architecture and function”, significantly advances our understanding of DNA methyltransferase 1 (DNMT1) in neural development by revealing a critical role in postmitotic SST-expressing cortical interneurons. Using conditional mouse genetics (*Sst-Cre/tdTom/Dnmt1 loxP*^2^ mice), live imaging, and functional assays, the authors demonstrate that DNMT1 regulates the expression of key transcription factors required for interneuron subtype identity and migration, such as *Arx*, in a DNA methylation-dependent manner at the postmitotic level. Loss of DNMT1 disrupts the directed migration of SST^+^ interneurons, causing premature cortical plate invasion. Notably, these alterations affect the surrounding microenvironment in a non-cell-autonomous fashion, impacting cortical progenitor proliferation and laminar organization. These alterations culminate in impaired cortical function and behavioral deficits related to neurological and neuropsychiatric diseases. Of note, the adult cortex of conditional mutants contained an increased fraction of SST^+^ interneurons co-expressing parvalbumin, suggesting a fate shift within this lineage. These findings not only highlight a previously underappreciated epigenetic safeguarding of interneuron trajectory and timing at the postmitotic level by DNMT1 but also open new avenues to explore how epigenetic regulators coordinate cellular interactions during corticogenesis—insights with potential relevance for neurodevelopmental disorders involving interneuron dysfunction.

These insights should be viewed within the broader context of epigenetic regulation in the nervous system. DNA methylation and chromatin remodeling are now recognized as dynamic, context-sensitive processes that shape cell identity, not only during early lineage commitment but also throughout maturation and integration into functional circuits (Luo et al., 2018). GABAergic interneurons exemplify this principle, given their extended period of postmitotic maturation, their subtype diversity, sensitivity to environmental factors, and their implication in neurodevelopmental and psychiatric disorders (Yildiz and Zimmer-Bensch, 2022). Epigenetic regulators such as DNMT1 are uniquely positioned to bridge intrinsic genetic programs and extrinsic environmental signals across developmental steps.

Molecular dynamic simulations presented by Reichard et al. (2025) provide compelling evidence that DNMT1 can function beyond its canonical role as a maintenance methyltransferase. While X-ray crystallography suggests weak binding of DNMT1 to unmethylated DNA (UMDNA) (Song et al., 2011), this contrasts with nanomolar-range dissociation constants measured in solution—comparable to those for hemimethylated DNA (Pradhan et al., 2008). This discrepancy likely stems from crystal packing artifacts that restrict UMDNA–protein interactions. To resolve this, the authors performed molecular dynamics simulations, revealing that UMDNA undergoes conformational rearrangements in solution that enable stable binding to catalytic domain of DNMT1, including key interactions at CpG sites (Reichard et al., 2025). Contact analysis showed a **~**60% increase in binding interfaces relative to the crystal structure, aligning with other high-affinity DNA–protein complexes (Reichard et al., 2025). These findings support an expanded role for DNMT1 that includes binding to unmethylated DNA with regulatory potential in postmitotic neurons, particularly where DNA replication–coupled maintenance methylation is absent.

Such non-canonical functions are consistent with earlier work showing migration deficits upon *Dnmt1* deletion in postmitotic, *Hmx3*-expressing POA-derived interneurons, and transcriptional dysregulation of cytoskeletal genes via crosstalk with histone modifications (reviewed in Yildiz and Zimmer-Bensch, 2022). Similarly, *Dnmt1* deletion in mature parvalbumin interneurons impairs synaptic transmission through altered endocytosis and vesicle recycling, underscoring a lifelong requirement for DNMT1 in maintaining interneuron function (Yildiz and Zimmer-Bensch, 2022). Together, these studies define DNMT1 as a multifunctional regulator that operates in both developmental and mature neuronal contexts through DNA-methylation–dependent and –independent mechanisms.

A central question is how DNMT1 coordinates such diverse functional modalities and target gene programs. Given that the transcriptional and epigenetic landscape of a neuron is shaped not only by its intrinsic state but also by extracellular signals (Zimmer-Bensch, 2018), epigenetic mechanisms are increasingly recognized as key integrators of environmental cues. Thus, DNMT1 may act as a dynamic integrator of environmental signals. During cortical interneuron migration and cortical circuit assembly, cells encounter membrane-bound guidance cues, neurotransmitters, and diffusible factors (Zimmer-Bensch, 2018), all capable of triggering intracellular pathways that remodel chromatin and gene expression. Increasing evidence shows that environmental factors, including neurotransmitters, hormones, and stress mediators, can reshape neuronal methylation landscapes and influence DNMT function (Brandão and Romcy-Pereira, 2015), particularly in GABAergic subtypes critical for cortical circuit formation and cognition.

Our recent work provided mechanistic insight into how such coupling can occur (Yildiz et al., 2023). EphrinA5 directs the subcellular targeting of DNMT1 via long non-coding RNAs (lncRNAs), thereby guiding chromatin remodeling in a context-dependent fashion. This ephrinA5–lncRNA–DNMT1 axis exemplifies how extracellular signals can be transduced into lasting transcriptional states in postmitotic neurons. Comparable modulation may arise from neuronal activity, neuromodulators (serotonin and dopamine), or steroid hormones (estradiol and corticosterone), known to modulate the DNA methylation machinery either directly or indirectly (Kundakovic and Champagne, 2015).

Beyond local signaling, DNMT1 expression and DNA-methylation signatures are sensitive to organismal environmental factors. Early-life stress, for example, alters methylation patterns in stress-responsive brain regions via glucocorticoid receptor–dependent mechanisms (Blaze et al., 2009). Similarly, perinatal exposure to endocrine disruptors and environmental toxins dysregulate DNMT1 and related DNA methylation machinery in the developing brain, being even related to neurodevelopmental disorders (Singh and Li, 2012; Kundakovic and Champagne, 2015). Viral infection can further reshape DNA methylation in an organoid model of the developing human brain (Janssens et al., 2018). Collectively, these observations position DNMT1 and other writers as molecular transducers at the intersection of intrinsic and extrinsic signaling, translating psychosocial, infectious, and chemical exposures into durable epigenetic adaptations.

Given the high sensitivity of cortical interneurons to developmental and environmental perturbations —and their implication in neurodevelopmental disorders such as schizophrenia, autism spectrum disorders, and epilepsy (Yildiz and Zimmer-Bensch, 2022) — such fine-tuned regulation of DNMT1 may be essential for developing and maintaining inhibitory circuit homeostasis.

These concepts align with the emerging view that interneuron identity and function are not exclusively determined at the progenitor stage but continue to be shaped by extrinsic and epigenetic inputs postmitotically. In this regard, a recent study (Bright et al., 2025) provides compelling evidence that maturation competence of interneuron progenitors, and their subsequent differentiation trajectories, is governed by temporally encoded signals that modulate chromatin accessibility and transcription factor engagement. These observations reinforce the concept that environmental modulation of the epigenome, including through DNMT1, may act beyond the progenitor stage to refine interneuron identity and integration.

In summary, DNMT1 emerges as a multifunctional regulator that operates at the intersection of intrinsic transcriptional networks and extrinsic environmental signals. By combining its classical DNA methylation activity with broader roles in chromatin and signal integration, DNMT1 contributes to both the temporal precision and spatial specificity of interneuron development. Its continued function in mature interneurons further suggests that DNMT1 may be central to maintaining circuit stability throughout life, where environmentally triggered stressors could impair its function and thereby contribute to brain dysfunction (**Figure 1**). Future studies should aim to dissect the upstream signals and downstream effectors that confer target specificity to DNMT1 in distinct interneuron subtypes and developmental windows. Understanding these pathways may ultimately reveal new therapeutic entry points for neurodevelopmental and neuropsychiatric disorders characterized by interneuron dysfunction.

**Figure 1:**
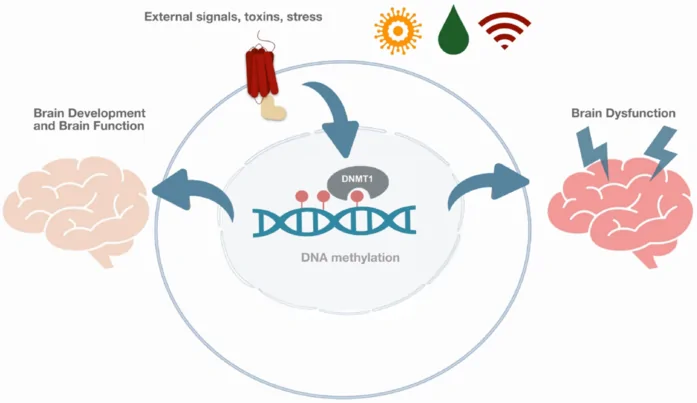
Extrinsic signals impact DNA methyltransferase 1 (DNMT1) function in the developing and adult brain. During brain development, DNMT1 controls proper temporal and spatial gene expression programs essential for neuronal differentiation and circuit formation. Throughout life, DNMT1 continues to maintain epigenetic stability in mature neurons, thereby supporting normal brain function. External signals such as stress, toxins, and inflammation can modulate DNMT1 activity, potentially disrupting its regulatory network and leading to maladaptive epigenetic changes associated with brain dysfunction. Created with Keynote and Affinity.

## References

[R1] Blaze J, Roth TL (2017). Caregiver maltreatment causes altered neuronal DNA methylation in female rodents. Dev Psychopathol.

[R2] Brandão JA, Romcy-Pereira RN (2015). Interplay of environmental signals and progenitor diversity on fate specification of cortical GABAergic neurons. Front Cell Neurosci.

[R3] Bright AR, Kotlyarenko Y, Neuhaus F, Rodrigues D, Feng C, Peters C, Vitali I, Dönmez E, Myoga MH, Dvoretskova E, Mayer C (2025). Temporal control of progenitor competence shapes maturation in GABAergic neuron development in mice. Nat Neurosci.

[R4] Janssens S, Schotsaert M, Karnik R, Balasubramaniam V, Dejosez M, Meissner A, García-Sastre A, Zwaka TP (2018). Zika virus alters DNA methylation of neural genes in an organoid model of the developing human brain. mSystems.

[R5] Kundakovic M, Champagne FA (2015). Early-life experience, epigenetics, and the developing brain. Neuropsychopharmacology.

[R6] Luo C, Hajkova P, Ecker JR (2018). Dynamic DNA methylation: in the right place at the right time. Science.

[R7] Murgatroyd C, Patchev AV, Wu Y, Micale V, Bockmühl Y, Fischer D, Holsboer F, Wotjak CT, Almeida OF, Spengler D (2009). Dynamic DNA methylation programs persistent adverse effects of early-life stress. Nat Neurosci.

[R8] Pradhan M, Estève PO, Chin HG, Samaranayke M, Kim GD, Pradhan S (2008). CXXC domain of human DNMT1 is essential for enzymatic activity. Biochemistry.

[R9] Reichard J (2025). DNMT1-mediated regulation of somatostatin-positive interneuron migration impacts cortical architecture and function. Nat Commun.

[R10] Singh S, Li SS (2012). Epigenetic effects of environmental chemicals bisphenol A and phthalates. Int J Mol Sci.

[R11] Song J, Rechkoblit O, Bestor TH, Patel DJ (2011). Structure of DNMT1-DNA complex reveals a role for autoinhibition in maintenance DNA methylation. Science.

[R12] Yildiz CB, Zimmer-Bensch G (2022). Role of DNMTs in the brain. Adv Exp Med Biol.

[R13] Yildiz CB, Kundu T, Gehrmann J, Koesling J, Ravaei A, Wolff P, Kraft F, Maié T, Jakovcevski M, Pensold D, Zimmermann O, Rossetti G, Costa IG, Zimmer-Bensch G (2023). EphrinA5 regulates cell motility by modulating Snhg15/DNA triplex-dependent targeting of DNMT1 to the Ncam1 promoter. Epigenetics Chromatin.

[R14] Zimmer-Bensch G (2018). Diverse facets of cortical interneuron migration regulation - implications of neuronal activity and epigenetics. Brain Res.

